# Infections Caused by Non-Tuberculous Mycobacteria in Recipients of Hematopoietic Stem Cell Transplantation

**DOI:** 10.3389/fonc.2014.00311

**Published:** 2014-11-10

**Authors:** Khalid Ahmed Al-Anazi, Asma M. Al-Jasser, Waleed Khalid Al-Anazi

**Affiliations:** ^1^Section of Adult Hematology and Oncology, Department of Medicine, King Khalid University Hospital, College of Medicine, King Saud University, Riyadh, Saudi Arabia; ^2^Central Regional Laboratory, Ministry of Health, Riyadh, Saudi Arabia; ^3^Section of Microbiology, Department of Pathology, King Khalid University Hospital, College of Medicine, King Saud University, Riyadh, Saudi Arabia

**Keywords:** non-tuberculous mycobacteria, hematopoietic stem cell transplantation, central venous catheters, colonization, combined antimicrobial therapy

## Abstract

Non-tuberculous mycobacteria (NTM) are acid-fast bacteria that are ubiquitous in the environment and can colonize soil, dust particles, water sources, and food supplies. They are divided into rapidly growing mycobacteria such as *Mycobacterium fortuitum, Mycobacterium chelonae*, and *Mycobacterium abscessus* as well as slowly growing species such as *Mycobacterium avium, Mycobacterium kansasii*, and *Mycobacterium marinum*. About 160 different species, which can cause community acquired and health care-associated infections, have been identified. NTM are becoming increasingly recognized in recipients of hematopoietic stem cell transplantation (HSCT) with incidence rates ranging between 0.4 and 10%. These infections are 50–600 times commoner in transplant recipients than in the general population and the time of onset ranges from day 31 to day 1055 post-transplant. They have been reported following various forms of HSCT. Several risk factors predispose to NTM infections in recipients of stem cell transplantation and these are related to the underlying medical condition and its treatment, the pre-transplant conditioning therapies as well as the transplant procedure and its complications. Clinically, NTM may present with: unexplained fever, lymphadenopathy, osteomyelitis, soft tissue and skin infections, central venous catheter infections, bacteremia, lung, and gastrointestinal tract involvement. However, disseminated infections are commonly encountered in severely immunocompromised individuals and bloodstream infections are almost always associated with catheter-related infections. It is usually difficult to differentiate colonization from true infection, thus, the threshold for starting therapy remains undetermined. Respiratory specimens such as sputum, pleural fluid, and bronchoalveolar lavage in addition to cultures of blood, bone, skin, and soft tissues are essential diagnostically. Susceptibility testing of mycobacterial isolates is a basic component of optimal care. Currently, there are no guidelines for the treatment of NTM infections in recipients of stem cell transplantation, but such infections have been successfully treated with surgical debridement, removal of infected or colonized indwelling intravascular devices, and administration of various combinations of antimicrobials. Monotherapy can be associated with development of drug resistance due to new genetic mutation. The accepted duration of treatment is 9 months in allogeneic stem cell transplantation and 6 months in autologous setting. Unfortunately, eradication of NTM infections may be impossible and their treatment is often complicated by adverse effects and interactions with other transplant-related medication.

## Introduction

Non-tuberculous mycobacteria (NTM) are ubiquitous environmental organisms that have generally been considered as an uncommon cause of human disease. Before the acquired immune deficiency syndrome (AIDS) epidemic, most cases presented as indolent, cavitating pulmonary infections in individuals with underlying lung disease such as chronic obstructive airway disease (COPD) or previous tuberculosis (1). During the 1980s, pulmonary and disseminated infections due to the more common NTM such as *Mycobacterium avium* (*M. avium*), *M. intracellulare, M. scrofulaceum* emerged as complications of AIDS and were termed as *M. avium* complex (MAC) infections (1).

The frequent isolation of NTM other than MAC from solid organ transplant (SOT) and hematopoietic stem cell transplantation (HSCT) recipients limits the extrapolation of therapeutic data from human immunodeficiency virus (HIV)-infected individuals to the population of transplant recipients (1). The spectrum of mycobacterial infections associated with SOT and HSCT differs from that associated with AIDS. Mycobacterial infections in transplant recipients tend to be more tissue-invasive (1).

## Microbiology

Non-tuberculous mycobacteria are aerobic, non-motile organisms that appear positive with acid-fast alcohol stains. The thick lipid-rich hydrophobic cell wall renders them impermeable to hydrophilic nutrients and resistant to heavy metals, disinfectants, and antibiotics (2). They form biofilms that contribute further to resistance to disinfectants and antibiotic therapy (2). Host factors organism characteristics and interferon-γ trafficking influence susceptibility and manifestations of NTM infection (2, 3). Pathogenic NTM are usually less virulent than *M. tuberculosis*. NTM may be isolated without having evidence of disease, but species of NTM that are usually considered contaminants may produce disease in immunocompromised individuals (3).

## Pathogenesis

The mode of transmission of NTM to humans is not well defined (2, 3). Aerosolization of small droplets is the likely route of acquisition of pulmonary disease. Contamination of hospital water supplies, medical equipment such as bronchoscopes and endoscopes and contamination of dialysis solutions has led to both NTM colonization and health care-associated outbreaks of infection (2). Environmental exposure plays a major role in transmission of NTM in immunocompromised hosts (3).

## Environmental Sources of NTM

Environmental sources of NTM include tap water, shower heads, soil and dust, food products in addition to domestic and wild animals. NTM are a miscellaneous collection of acid-fast bacilli (AFB) that are widely spread in the environment (2, 4–6). More than 160 NTM species have been identified, but not all of them cause human disease (2, 6, 7).

## Classification of NTM Species

Historically, different classification systems have been proposed including (1) atypical mycobacteria, (2) mycobacteria other than tuberculosis, and (3) NTM. The last classification (NTM) is the currently used one and NTM species are usually classified into slowly growing or rapidly growing NTM (2, 3).

Rapidly growing NTM include *M. fortuitum, M. chelonae, M. abscessus, M. neoaurum, M. flavescens, M. mucogenicum*, and other species (2, 6–9). They grow within 7 days and they have been associated with infections in SOT recipients more than in HSCT patients (6, 10). They may be susceptible to the following antimicrobials: (1) macrolides such as azithromycin and clarithromycin, (2) aminoglycosides such as amikacin and tobramycin, (3) tetracyclines such as doxycycline and monocycline, (4) fluoroquinolones such as ciprofloxacin and levofloxacin, (5) linezolid, (6) tigecycline, (7) imipenem, (8) cefoxitin, and (9) trimethoprim–sulfamethoxazole (TMP–SMZ) (3, 8, 9).

Slowly growing NTM require several weeks to grow and they include MAC, *M. kansasii, M. marinum, M. ulcerans, M. xenopi, M. simiae, M. szulgi, M. genavense, M. gordonae, M. haemophilum*, and *M. malmoense* in addition to many other species (2, 6, 7). MAC complex includes *M avium, M. intracellulare, M. silvaticum, M. hominissius, M. paratuberculosis*, and many others (2). MAC is a common cause of pulmonary and disseminated NTM infections (2, 10). *M. kansasii* is the second most common cause of pulmonary infections caused by NTM in United States of America (USA) and England, the commonest being MAC (2). Slowly growing NTM may be susceptible to the following antimicrobials: isoniazid (INH), rifampicin and rifabutin, ethambutol, aminoglycosides such as amikacin and streptomycin, macrolides such as ciprofloxacin and levofloxacin, quinolones, and sulfamethoxazole (3).

## Epidemiology and Significance of Isolation of NTM

Over the past three decades, the global prevalence of pulmonary NTM disease has increased dramatically (11). In North America, the rates of pulmonary NTM disease and infection range from 1 to 15 per 100,000 and 0.1 to 2 per 100,000 population, respectively. In Europe, the incidence rates are generally similar to those in North America, with the exception of high rates in mining communities in the Czech Republic (12). Epidemiological evaluation of NTM infection is more challenging than that of tuberculosis due to the following reasons: (1) lack of systemic reporting of NTM infections in most countries, (2) NTM disease rates vary considerably depending on the population and the geographic location, (3) as NTM are soil and water organisms, infection is thought to be acquired from environment rather than transmitted from person to person, and (4) colonization of respiratory tract can occur without causing pulmonary tuberculosis (11, 12). Only 6–25% of NTM isolates obtained from various clinical specimens have been considered clinically significant according to three major studies performed in the USA, Switzerland, and South Korea (13–15). In the absence of mandatory reporting of infections, the true incidence of NTM disease in transplant recipients and in the general population can be only estimated (1). The epidemiology of NTM infections in patients without AIDS remains somewhat difficult to be determined as clinically significant colonization can be difficult to be distinguished from true infection (16). Although the isolation of NTM from clinical specimens has recently increased, laboratory-based surveillance can produce reasonable estimates of the real incidence of NTM disease (16). Therefore, large multicenter regional studies and mandatory reporting will be required to better understand the changing epidemiology of NTM infections in patients without AIDS (16).

## Risk Factors for NTM Infections

Risk factors for NTM infections in the general population are very variable and they are listed in Table 1 (5–9, 17–32).

**Table 1:** **Shows risk factors for NTM infections in the general population**.

(1) Advanced human immunodeficiency virus infection
(2) Presence of comorbid medical conditions such as
**Cystic fibrosis
**Idiopathic pulmonary fibrosis
**Coal workers’ pneumoconiosis
**Rheumatoid arthritis
**Previous pulmonary tuberculosis infection
(3) Congenital disorders including specific deficiencies and genetic mutations such as
**Interferon-γ receptor 1 and 2 deficiencies
**Interleukin-12 deficiency
**Signal transduction and activator of transcription-1 deficiency
**Interferon regulator factor 8 deficiency
**GATA-2 (mono-MAC syndrome) deficiency
**Nuclear factor kappa-β essential modulator deficiency
(4) Old age
(5) Male sex
(6) Warm climate
(7) Solid tumors and hematologic malignancy
(8) Cytotoxic chemotherapy or radiotherapy administered to control malignancy
(9) Immunosuppressive therapies including alemtuzumab, rituximab, and infliximab
(10) Solid organ and stem cell transplantation
(11) Intravascular indwelling catheters, particularly in immunocompromised individuals
(12) Neutropenia

## High-Risk Groups for NTM Infections

Non-tuberculous mycobacteria infections are increasingly reported in SOT and HSCT recipients. Causes of increased incidence of NTM infections in transplant recipients include increase in the number of transplants done worldwide, intensification of immunosuppressive therapies, prolonged survival of transplant patients, and improvements in diagnostic techniques (1). Treatment of NTM infections in transplant recipients comprises reduction in immunosuppressive therapy, antimicrobials, and surgical debridement, if needed (33–37).

Non-tuberculous mycobacteria infections have been reported in patients with hematological malignancy and in those with solid tumors. The risk factors for NTM infections in cancer patients include (1) the primary disorder, (2) cytotoxic chemotherapy, (3) the recent use of monoclonal antibodies and targeted therapies such as rituximab and alemtuzumab, and (4) HSCT in patients with hematological malignancy (17–19, 21, 28). In such patients, antimicrobial treatment can be administered without necessarily compromising cancer therapy (17). Surgical treatment may be in the form of surgical resection of a lung segment having a cavity or debridement of an infected CVC tunnel or a deep seated soft tissue infection (17–19, 21).

## Clinical Aspects of NTM Infections

Infections caused by NTM may present with (1) lymphadenopathy, (2) skin and soft tissue infections, (3) pulmonary disease, particularly in patients with underlying lung disease, or (4) disseminated disease, particularly in immunocompromised individuals (2, 5–7). Clinical manifestations and complications of NTM infections vary according to the species isolated and the site of involvement and they include fever, weight loss, abdominal pain, diarrhea, dyspnea, productive cough, chest pain, lymphadenopathy, hepatosplenomegaly, and infections involving CVCs, skin and soft tissues, genital tract, and central nervous system (6, 18, 37, 38). At times, NTM infections may be entirely asymptomatic (18, 37, 38).

## Disseminated NTM Infections

Disseminated NTM infections occur almost exclusively in immunocompromised hosts (6, 7, 18, 19). MAC is the most common NTM species associated with disseminated infections (6, 18, 19). Disseminated NTM infections involve bone marrow, lymphoreticular system, gastrointestinal (GTI) tract, lungs, and skin (6, 18, 19). Clinical manifestations of disseminated NTM infections include fever, weight loss, sweating, diarrhea, generalized lymphadenopathy, disseminated skin lesions, diffuse abdominal tenderness, and hepatosplenomegaly (6).

Laboratory finding in disseminated NTM infections include (1) normocytic anemia, (2) bacteremia that may be intermittent or low-grade, and (3) elevated serum levels of lactic dehydrogenase and alkaline phosphatase (6). The differential diagnosis of disseminated NTM infections includes (1) disseminated *M. tuberculosis* infection, (2) disseminated fungal infections such as candidiasis or invasive aspergillosis, (3) metastatic malignancy, (4) actinomycosis, and (5) nocardiosis (6). Disseminated NTM disease should be suspected in immunocompromised hosts presenting with non-specific clinical manifestations such as fever, weight loss, and abdominal pain or skin lesions without other possible explanations (6).

Treatment of disseminated NTM infections is usually undertaken in consultation with an expert in infectious diseases (6). If NTM bacteremia is associated with infection of CVC, removal of the catheter is strongly recommended. Recovery of the immune system improves survival in patients with disseminated NTM disease (6). Medical therapy of disseminated NTM infections and NTM bacteremia includes initial and continuation phases (6). In the initial phase, antimicrobials are usually given for 1–2 months or till clinical or radiological improvement is achieved. At least three drugs to which the NTM species isolated is susceptible should be administered. Typical regimens include (a) macrolides such as clarithromycin or azithromycin, (b) ethambutol, and (c) rifampicin or rifabutin (6). Randomized observational studies in HIV patients with MAC infections have shown that multidrug regimens that include macrolides are associated with clinical and microbiological improvements (6). The addition of rifampicin to multidrug regimens has been associated with shorter duration of bacteremia and decreased level of macrolide resistance (6). The continuation phase starts once the patient has demonstrated clinical improvement. The antimicrobial regimen is usually composed of two drugs and the total duration of therapy for disseminated NTM infection or NTM bacteremia is usually 6–12 months (6). Antimicrobial prophylaxis may prevent disseminated disease in immunocompromised individuals, but despite treatment, disseminated NTM infections carry substantial morbidity and mortality (6).

## Diagnosis of NTM Infections

Diagnosis of NTM disease in recipients of SOT and HSCT is usually difficult. To ensure prompt diagnosis and early initiation of therapy, a high index of suspicion for NTM disease should be maintained (1). Definitive diagnosis of NTM disease requires recovery of NTM species from: blood, bone marrow, lymph nodes, liver, lung, and other body tissues or fluids such as pleural fluid (6). However, culture is essential to differentiate NTM from *M. tuberculosis*, determine the species of NTM causing infection, and perform drug susceptibility testing (6).

Diagnostic tests for CVC-related NTM infections include blood cultures, cultures of swabs taken from exit sites of CVCs, cultures of swabs taken from discharge obtained from infected tunnels, and cultures taken from tips of intravascular catheters after removal (1).

Biopsy material taken from skin, pleural, and lung lesions should be subjected to histopathological examination, special stains, and microbiological cultures (1). The American Thoracic Society (ATS) and the Infectious Diseases Society of America (IDSA) diagnostic criteria for NTM pulmonary disease include (1) clinical: presence of clinical manifestations of pulmonary involvement, presence of underlying medical condition such as cancer or tuberculosis and appropriate exclusion of other diagnoses, (2) radiological: presence of pulmonary infiltration, nodular lung lesions, cavity formation or multifocal bronchiectasis on chest X-ray or high-resolution CAT scan, and (3) bacteriological: ≥2 positive cultures from expectorated sputum or ≥1 positive BAL or bronchial wash cultures or transbronchial or open lung biopsy showing histological evidence of granulomatous inflammation or AFB and positive NTM cultures or biopsy showing mycobacterial histological features and positive cultures for NTM from ≥1 sputum or bronchial washings (3, 39). It is essential to perform (1) cultures for other bacteria, *M. tuberculosis*, fungi, and *Nocardia* species as similar infections can be caused by these microorganisms in immunocompromised individuals, and (2) susceptibility testing of the mycobacterial isolates in order to have optimal care (1). Recommended susceptibility tests for rapidly growing and slowly growing NTM species are shown in Tables 2 and 3 (2, 3, 39–47).

**Table 2:** **Shows susceptibility testing and therapeutic agents in rapidly growing NTM isolates**.

Organism	Recommended susceptibility testing			Effective therapeutic agents		
*M. abscessus*	*Macrolides: clarithromycin/azithromycin			**Macrolides (clarithromycin or azithromycin) with 1 or 2 other agents		
	*Quinolones: ciprofloxacin, levofloxacin, moxifloxacin			**In case of macrolide resistance		
	*Aminoglycosides: tobramycin, amikacin			-Quinolones: ciprofloxacin, levofloxacin, moxifloxacin		
	*Tigecycline	*Cefoxitin	*Clofazimine	- Amikacin	- Cefoxitin	- Imipemem
	*Linezolid	*Imipenem	*Aztreonam	- Linezolid	- Tigecycline	

*M. chelonae*	*Aminoglycosides: tobramycin, amikacin			*Macrolides (clarithromycin or azithromycin) with 1 or 2 additional agents with *in vitro* susceptibility		
	*Macrolides: clarithromycin			*Aminoglycosides: tobramycin, amikacin		
	*Quinolones: ciprofloxacin			*Quinolones: ciprofloxacin, moxifloxacin		
	*Tetracyclines: minocycline, doxycycline			*Linezolid	*Imipenem	*Tigecycline/doxycycline
	*Linezolid	*Imipenem	*Clofazimine	

*M. fortuitum*	*Macrolides: azithromycin			*Macrolides: clarithromycin, azithromycin		
	*Quinolones: ciprofloxacin, ofloxacin, moxifloxacin			*Quinolones: ciprofloxacin, levofloxacin, moxifloxacin		
	*Tetracyclines: monocycline, doxycycline, minocycline			*Tetracyclines: doxycycline, tigecycline		
	*Aminoglycosides: tobramycin, amikacin			*Linezolid	*Cefoxitin	*TMP–SMZ
	*Sulfamethoxazole	*Imipenem		**Two agents with *in vitro* susceptibility should be used		
	*Cefoxitin	*Cephalexin	

*M. neoarum*	*Aminoglycosides: tobramycin, amikacin			**Aminoglycosides: tobramycin/amikacin		
	*Tetracyclines: doxycycline, minocycline			**Quinolones: ciprofloxacin, levofloxacin, moxifloxacin		
	*Macrolides: clarithromycin			**Macrolides: clarithromycin, azithromycin		
	*Quinolones: ciprofloxacin			**Tetracyclines: doxycycline, tigecycline		
	*Linezolid	*Cefoxitine		**Linezolid	**Cefoxitin
	*Imipenem	*Cotrimoxazole		**Imipenem	**TMP–SMZ

*M. mucogenicum*	Susceptibility testing should be performed for the following antimicrobials					
	*Amikacin	*Clarithromycin	*Doxycycline	*Ciprofloxacin	*Linezolid
	*Tobramycin	*TMP–SMZ	*Minocycline	*Imipenem	*Cefoxitin

*NTM, non-tuberculous mycobacteria; TMP/SMZ, trimethoprim–sulfamethoxazole*.

**Table 3:** **Shows recommended susceptibility testing and active therapeutic agents used in the treatment of slowly growing NTM isolates**.

Organism	Recommended susceptibility testing			Effective therapeutic agents		
*M. kansasii*	**Rifampin susceptibility should be performed for all new/untreated *M. kasasii* isolates			**Combination therapy: daily or three times per week		
	***In case of rifampin resistance			- Rifampin/rifabutin	- Isoniazid - Ethanbutol
	**Quinolones			**In case of Rifampin resistance		
	**Clarithromycin	**INH	**Streptomycin	A- Macrolide + quinolone based therapy: clarithromycin/azithromycin, moxiflxacin, linezolid		
	**Sulfamethoxazole	**Ethambutol	**Amikacin	B- High-dose isoniazid +1 or 2 other agents: Quinolone, macrolide, aminoglycoside, TMP/SMZ or linezolid		

MAC	*Routine susceptibility testing is not performed in most laboratories except in case of clarithromycin resistance			**Limited disease: azithromycin/clarithromycin + rifampin/rifabutin+ethambutol		
	**Clarithromycin	**Rifabutin		**Extensive/cavitary disease: above regimen+aminoglycoside: streptomycin/amikacin		
	**Quinolones	**Ethambutol		**Macrolide resistance: isoniazid, rifabutin, ethambutol, macrolide, linezolid, streptomycin/amikacin		

*M. szulgai*	**INH	**Aminoglycosides		**Isoniazid, rifampin, ethambutol ? 4th drug such as pyrazinamide		
	**Rifampin	**Quinolones		
	**Ethambutol	**Macrolides		

*M. xenopi*	**INH			**Isoniazid, rifampin/rifabutin ? streptomycin		
	**Rifampin	**Quinolones		**Clarithromycin + rifampin + ethambutol		
	**Ethambutol	**Macrolides		**Other active agents: clarithromyci/azithromycin, amikacin, moxifloxacin		

*M. malmoense*	**INH			**Isoniazid, rifampin + ethambutol ? macrolide and/ or quinolone		
	**Ethambutol	**Quinolones		
	**Rifampin	**Macrolides		

*M. marinum*	*M. marinum isolates do not require susceptibility testing unless therapy fails for several months			**Clarithromycin or azithromycin + ethambutol		
	**Doxycycline	**Ethambutol		**Surgical debridement	**Rifampin in case of osteomyelitis
	**Rifampin	**Sulfonamides		**Other active agents: ethambutol, TMP/SMZ, rifampin, rifabutin, ciprofloxacin, moxifloxacin, amikacin, doxycycline		

*M. haemophilum*	**Aminoglycosides: streptomycin/amikacin			**At least three drugs	**Variable duration: 3–42 months	
	**Quinolones: ciprofloxacin			**Active drugs: amikacin, rifampin, rifabutin, ethambutol, clarithromycin, ciproflxacin		
	**Macrolides: clarithromycin			

*M. ulcerans*	*Medical therapy has not been shown to be beneficial		**Rifampin	**Moxifloxacin		
			**Dapsone	**Diarylquinolone R 207910		
	*Surgical Rx with skin grafting are vital		**Streptomycin	**Nitroimidazopyran PA-824		
	*Combinations of *in vitro* active agents may be beneficial		**Amikacin	**Linezolid		

The use of 16S rRNA sequencing has been introduced for the identification of mycobacterial species including NTM (48). It has a number of advantages including marked shortening of the turnaround time, and identification of mycobacterial strains with ambiguous biochemical and fatty acid profiles. The recent increase in the utilization of 16S rRNA sequencing in the identification of NTM isolates may render its use cost effective (48).

Multiple blood cultures are needed because NTM bacteremia may be intermittent or low grade (6). Additionally, mycobacterial blood cultures are usually collected in special media, different from the media used in standard bacterial blood cultures and must be specifically requested. However, blood cultures may be negative even in disseminated NTM disease (6).

In the presence of negative blood cultures, tissue biopsies become essential diagnostically. Such biopsies that are usually obtained from bone marrow, liver, skin, lungs, and lymph nodes are needed for histopathology, acid-fast stains and cultures (6). Histopathological changes in various forms of NTM disease are shown in Table 4 (49–51). Tissue biopsies demonstrating AFB or granuloma formation strongly support the diagnosis of disseminated NTM infection in the presence of negative blood cultures (6). Tissue biopsies taken from immunocompromised adults with disseminated NTM infection show 50% of patients have biopsy findings consistent with mycobacterial granulomas and 20% have positive AFB smears (6).

**Table 4:** **Shows histopathological changes in various forms of NTM infection/disease**.

NTM cutaneous lesions	NTM lymphadenitis	NTM lung lesions
*Neutrophil infiltration	**Microabscesses	Three distinct patterns
*Interstitial granuloma	**Ill-defined non-caseating granulomas	(1)Granulomas with variable degrees of necrosis
*Small vessel proliferation	**Few giant cells	(2)Non-caseating epitheloid cell granulomas
*Increased numbers of acid-fast bacilli		(3) Interstitial fibrosis and organizing pneumonia

*NTM, non-tuberculous mycobacteria*.

The rapid increase in identified NTM species in recent years reflects not only improved culturing techniques but also more precise differentiation of species (2). Species differentiation has improved dramatically with the development of molecular techniques that allow detection of differences in 16S rRNA gene, which is highly conserved amongst species though slight differences characterize different species (2). More rapid diagnosis of NTM and faster differentiation from *M. tuberculosis* can be attributed to the introduction of BACTEC fluid culture systems and the development of nucleic acid amplification tests (NAAT) and DNA probe techniques (5). Also, identification of new NTM species has been facilitated by (1) high-performance liquid chromatography, (2) polymerase chain reaction (PCR), and (3) restriction fragment length polymorphism analysis (2).

## Treatment of NTM Infections

Therapeutic regimens are difficult to adhere to because of long duration of treatment, adverse events and drug interactions with other drugs that patients require. New therapeutic regimens that include newer macrolides are more effective than old regimens, but failure rates are still very high and relapses may occur even after apparently successful therapy (5). Recommended therapeutic regimens and antimicrobial agents that are active against rapidly growing as well as slowly growing NTM species are shown in Tables 2 and 3 (2, 3, 39, 40, 42, 44, 45, 52–57). The evolution of resistance to antimicrobials used in the therapy of NTM infection is a real concern as drug resistance increases the chances of treatment failure. Mechanisms of drug resistance in NTM are shown in Table 5 (40, 47).

**Table 5:** **Shows mechanisms of drug resistance in NTM**.

(1) Efflux pumps
(2) Selective permeability of cell wall
(3) Lack of prodrug activation
(4) Plasmid-mediated drug resistance
(5) Antibiotic inactivation by antibiotic modifying or degrading enzymes
Examples: [a] Broad spectrum β-lactamases [b] Aminoglycoside modifying/degrading enzymes
(6) Target site alterations
[a] Ribosomal protection [b] Genetic mutations and polymorphisms
Examples; mutations in
• erm gene • 16 S r RNA
• rps L gene • 23 S r RNA
• emb B gene • DHPSH: involved in folate metabolism
• kat G gene • DHFR: involved in folate metabolism
• inh A gene

## Other Adjunctive Therapies for NTM

Refractory and disseminated infections caused by slowly growing and rapidly growing NTM have been reported to be successfully treated with interferon-γ, interferon α 2b, and rituximab (58–61).

## Prognosis of NTM Infections

A population-based comparative study performed in USA between 1999 and 2010 showed that the number of deaths from NTM disease was rising particularly in individuals ≥55 years, women, white ethnicity and those living in certain locations such as Louisiana and Hawaii and that the risk factors for mortality included COPD, bronchiectasis, interstitial lung disease, HIV, and tobacco use (62). The outcome of NTM infections is generally good for localized infection and is often poor for disseminated disease (33–37). Eradication of NTM infection is usually difficult and recurrent infection with new strains of NTM or even relapse of infection caused by the original organism is not uncommon (2).

## NTM Infections in Recipients of HSCT

Hematopoietic stem cell transplantation is being used to treat a wide spectrum of clinical disorders, but its use is limited by development of opportunistic infections, which carry significant morbidity and mortality (63). Recipients of HSCT have severely impaired cell mediated immunity as a consequence of underlying disease, pre-transplant chemotherapy, and radiotherapy in addition to graft versus host disease (GVHD) and its treatment (64).

Distinguishing infection from colonization by NTM such as MAC can be difficult as infection by microorganisms other than NTM and as non-infectious conditions, such as GVHD of lungs, may mimic NTM infections following HSCT (4, 65, 66). Patients with probable or possible NTM infection usually have different epidemiology, risk factors, sites of infection, species of NTM involved and prognosis from patients having definite infections (4, 66).

## CDC Definitions of NTM Disease in Allogeneic HSCT

Recently, centers for disease control and prevention (CDC) has provided criteria to define NTM infections in recipients of HSCT as follows:

### Lower respiratory tract

Criteria for definite NTM disease include (A) + (B) + (C), i.e., having (A) symptomatic patient with radiological evidence of disease (pneumonic infiltration, nodules, cavity formation, and bronchiectasis), (B) at least one positive culture of NTM from lower respiratory tract (BAL or deep tracheal sputum), and (C) one concomitant blood culture for NTM or histological evidence of tissue invasion by NTM (4, 66). Criteria for probable NTM infection include (A) + (B), i.e., the same criteria for definite NTM disease but without concomitant blood culture for NTM or histological evidence of tissue invasion by NTM (4, 66). Criteria for possible NTM infection include (A) + (B) + (D) or (E), i.e., the same criteria for probable NTM disease in addition to either (D) identification of a co-pathogen likely to account for the clinical picture, or (E) improvement of respiratory symptoms or pulmonary infiltration after treatment of an alternative diagnosis without specific treatment against NTM (4, 66).

### Catheter-related infections

Definite NTM infection is defined as having typical symptoms such as exit site or tunnel erythema, purulence or fever and a positive blood culture for NTM. However, probable NTM infection is defined as having typical symptoms and isolation of NTM from the catheter site, tunnel, or tip (4, 66).

### NTM infection involving other sites

Criteria for definite NTM infection include (A) + (B) + (C), i.e., having (A) compatible symptoms, (B) a positive culture of NTM from a usually sterile site, and (C) histological evidence of atypical mycobacterial tissue invasion or a positive blood culture. Criteria for probable NTM infection include (A) + (B), i.e., (A) presence of compatible symptoms, and (B) a positive culture of NTM from a normally sterile site, but no histological evidence of atypical mycobacterial tissue invasion or positive blood cultures (4, 66).

## Epidemiology of NTM Infections in HSCT

The incidence of NTM infections in adult recipients of HSCT ranges between 0.4 and 4.9% (1, 10, 65, 66). However, in allogeneic HSCT, this incidence may reach 9.7% (4, 5, 8, 63). NTM infections are increasingly reported in recipients of HSCT (63). The incidence of NTM infections in HSCT patients is 50–600 times greater than in general population (1). In pediatric recipients of HSCT, the incidence of NTM infections is less frequent than in adults receiving HSCT. The incidence of NTM infections in children receiving HSCT is about 3.8%, but has recently reached 6.4% in allogeneic setting (63). The recent increase in the incidence of NTM disease in pediatric recipients of allogeneic HSCT appears to be associated with the increased use of anti-T-cell antibodies such as ATG and alemtuzumab (63).

Non-tuberculous mycobacteria infections in HSCT recipients occur at a median of 115–1055 days post-HSCT (63, 66, 67). However, NTM infections have been reported as early as day 7 post-HSCT and have also been reported prior to HSCT (66–68). The incidence of NTM infections in recipients of HSCT varies from one transplant center to another (4). In T-cell depleted allografts, the incidence of NTM infections ranges between 0.37 and 9.7% (4, 66, 68). A combination of host, environmental, nosocomial and historical factors may contribute to the epidemiology of NTM disease in recipients of HSCT (4).

## Risk Factors for Development of NTM Infections in HSCT

The risk factors for development of NTM infections in recipients of HSCT are very variable and they are included in Table 6 (4, 26, 64–70). NTM infections have been reported more frequently in recipients of: allografts than in autografts, myeloablative transplants than in non-myeloablative transplants, and matched unrelated donor as well as mismatched allografts than in sibling allogeneic transplants (4, 65–71). NTM infections have been reported in patients receiving conditioning therapies comprising: total body irradiation, cyclophosphamide, busulphan, ATG, and alemtuzumab (4, 26, 64–70). NTM infections have been reported in almost all type of HSCT including: autologous HSCT, sibling allogeneic HSCT, unrelated allografts, umbilical cord blood transplants, T-cell depleted grafts, and myeloablative as well as non-myeloablative allogeneic transplants (4, 65–71). With better *M. tuberculosis* prophylaxis, intensive immunosuppressive therapy and better awareness, NTM infections have become an emergent marker of severe immunosuppression and poor prognosis. When there is doubt over species identity or extent of infection, broad spectrum antimicrobial cover may become prudent (65).

**Table 6:** **Shows risk factors for NTM infections in recipients of HSCT**.

(1) The type of HSCT
**Allogeneic more than autologous
**Mismatched or matched unrelated allografts more than sibling grafts
(2) The conditioning therapy used
**Myeloablative more than non-myeloablative allografts
(3) Use of T-cell depletion in allografts
(4) Use of alemtuzumab or anti-thymocyte globulin in conditioning therapies
(5) Acute or chronic GVHD
(6) Use of steroids in the treatment of GVHD
(7) Bronchiolitis obliterans and/or organizing pneumonia
(8) Having relapsed primary hematological disorder or malignancy prior to HSCT
(9) Pre-HSCT cytotoxic chemotherapy and radiotherapy
(10) Other related risk factors
**CVC
**Neutropenia
(11) Presence of other comorbid medical conditions

*NTM, nontuberculous mycobacteria; CVC, central venous catheter; HSCT, hematopoietic stem cell transplantation; GVHD, graft versus host disease*.

## NTM Species Reported in HSCT

In recipients of HSCT, the following rapidly growing NTM species have been reported to cause infections: *M. fortuitum, M. abscessus, M. chelonae*, and *M. mucogenium* (4, 64–68). The following slowly growing NTM species have also been reported to cause infections in recipients of various types of HSCT: *M. avium* and *M. intracellulare, M. kansasii, M. marinum, M. gordonae, M. haemophilum*, and *M. scrofulaceum* (4, 26, 63, 65–72). However, the most frequently isolated NTM species in recipients of HSCT are MAC, *M. haemophilum, M. gordonae, M. fortuitum, M. abscessus*, and *M. chelonae* (10, 66). NTM species may coexist with other organisms as causes of infections in recipients of HSCT such as: *Aspergillus fumigatus, Candida albicans, cytomegalovirus, respiratory syncytial virus, Pseudomonas aeruginosa*, and *Staphylococcus aureus* (64, 65, 67, 68).

## Specific Infections Caused by NTM in Recipients of HSCT

Non-tuberculous mycobacteria species have been reported to cause a variety of infectious complications in recipients of HSCT. These infectious complications include CVC-related infections, pleuropulmonary infections, skin and soft tissue infections, bloodstream infections, disseminated infections, GIT infections, bone marrow involvement, bone and joint infections in addition to involvement of liver and sinuses (4, 63, 65, 68–72).

## CVC-Related Infections Caused by NTM in HSCT

In recipients of HSCT, catheter-related infections are the most commonly encountered NTM infectious complications followed by pulmonary and cutaneous infections (1, 5, 8, 10). CVC-related infections account for 36% of all NTM infections in recipients of HSCT. These infections are often caused by rapidly growing NTM species (5). Slowly growing NTM species such as *M. gordonae* are a rare cause of CVC-related infections in recipients of HSCT (65).

The median time to diagnosis of CVC-related NTM infection from HSCT is approximately 61 days (66). The following NTM species have been reported to cause CVC-related infection in patients with HSCT: *M. fortuitum, M. chelonae, M. abscessus, M. mucogenicum*, and *M. neoaurum* (8, 9, 65, 66, 68). The clinical manifestations and complications of CVC-related NTM infections include exit site infection, tunnel infection, NTM bacteremia, and bloodstream infections in addition to disseminated disease (8, 9, 65, 66, 68). NTM bloodstream infections in recipients of HSCT are almost always associated with infections affecting indwelling intravascular catheters. However, disseminated NTM infections have been reported up to one year following initial CVC-related infection (8). Treatment of uncomplicated CVC-related NTM infections in transplant recipients includes removal of CVC, antimicrobial therapy and surgical debridement of soft tissues surrounding the tunnel of the affected CVC. The following antimicrobials can be used: ciprofloxacin, clarithromycin, doxycycline, amikacin, meropenem, and linezolid. Antimicrobial therapy should be guided by antimicrobial susceptibility testing results (8, 9, 66). A combination of two to three antimicrobials is usually required. Exit site infections can be treated for 3 weeks, while tunnel infections and NTM bloodstream infections require antimicrobial therapy for 6 weeks (8, 9, 66).

Complicated CVC-related infections caused by NTM and manifested by disseminated disease or bloodstream infection usually require (1) combination of antimicrobials depending on the identified NTM species and susceptibility testing results, (2) initially, broad and empirical antibiotic therapy can be administered in severely ill or immunocompromised individuals till identification and susceptibility results are obtained, then antimicrobial therapy may be narrowed accordingly, and (3) prolonged duration of therapy is usually required for recipients of HSCT: 6 months for autologous HSCT and 9 months for allogeneic HSCT (8). CVC-related NTM infections in HSCT patients usually have good prognosis, provided CVCs are removed early and appropriate antimicrobials are given. However, CVC-related infections may be complicated by disseminated infections despite antimicrobial treatment in severely immunocompromised hosts (8, 9, 66).

## Pulmonary Infections Caused by NTM in HSCT Recipients

Pleuropulmonary infections in HSCT recipients are usually caused by slowly growing NTM species with MAC being the most frequently encountered species (5, 66, 69). Pulmonary infections account for about 30% of all infections caused by NTM in recipients of HSCT (5, 66). Pulmonary infections can be a complication of CVC-related NTM infections (8). They can present with lung nodules or necrotizing pneumonia (8, 69). Chest X-rays and CAT scans of lungs are essential diagnostically (4, 8, 65–67). Radiological manifestations are variable and they include consolidation or patchy infiltration, pulmonary nodules that may be multiple, lung cavitations, multifocal bronchiectasis and pleural effusions (4, 8, 65–67). Cultures of sputum, BAL, pleural biopsy, and lung biopsy can be used to increase diagnostic yield. In addition, histopathology of lung biopsies may show granuloma formation in pulmonary infections caused by NTM in HSCT recipients (65, 66).

The following antibiotics can be given: azithromycin, clarithromycin, ciprofloxacin, and amikacin. Additionally, first line anti-tuberculosis drugs such as INH, ethambutol, rifampicin, and pyrazinamide can be given (8, 65, 66, 68). Duration of treatment is as follows: (1) a single agent can be given for 9 months, (2) a combination of 2 drugs can be given for 4–6 months, and (3) for complicated NTM infections, particularly in immunocompromised individuals, antimicrobial therapy can be continued for 12 to18 months (8, 65, 66, 68).

## Skin Infections Caused by NTM in HSCT Recipients

Skin infections due to NTM have been reported in recipients of HSCT. In the absence of appropriate therapy, localized infections may become disseminated and may even cause regional and painful lymphadenopathy in immunocompromised HSCT patients. However, even in the presence of targeted therapy, skin infections may disseminate (70, 71).

## Other Infections Caused by NTM in HSCT Recipients

Gastrointestinal involvement has been reported in HSCT patients. Other infections such as: bone, bone marrow, vertebrae, liver, maxillary sinus involvement and even lymphadenopathy with necrotizing granulomas have been reported in HSCT patients (4, 66, 68, 72). Disseminated NTM infections have also been reported in recipients of HSCT (66, 70–72).

## HSCT in Patients Previously Infected with NTM

Having NTM infection prior to transplant is not a contraindication to successful HSCT, as HSCT has been successfully performed in patients with history of treated NTM infections (24, 73). However, such patients may need to have anti-mycobacterial prophylaxis against NTM till day 100 post-HSCT (73).

## Diagnosis of NTM Infections in HSCT Recipients

Swabs taken from skin, subcutaneous tissue and nasal wash can be cultured for NTM. Also, NTM cultures can be taken from blood, skin lesions, pleural fluid, and BAL fluid. Susceptibility testing should be performed on positive cultures and biopsies can be taken from involved sites or organs. Skin biopsies may reveal granulomas on histology and may show evidence of AFBs (4, 64–66, 68–70). However, DNA hybridization methods may be needed for identification of NTM species. Biopsy material taken from lung, liver, bone marrow, and even vertebrae may show histopathological appearances consistent with NTM disease. Radiology is also helpful in diagnosis of NTM infections. Chest X-rays, CAT scans of chest, abdomen and pelvis may show the extent of NTM infection. Rates of positive NTM cultures vary from one transplant center or from one institution to another (4, 64–66, 68–70).

## Treatment of NTM Infections in Recipients of HSCT

Treatment of NTM infections in HSCT patients comprises medical therapy, surgical treatment and additional forms of therapy. Medical therapy is based on antimicrobials that are usually given in two to four drug combinations (63–66, 68–70). The following drugs are usually utilized in the treatment of NTM infections in HSCT recipients: aminoglycosides such as amikacin and tobramycin, macrolides such as minocycline, TMP–SMZ, ciprofloxacin, imipenem, linezolid, and tigecycline. Antimicrobial therapy should be based on results of antimicrobial susceptibility testing. Duration of therapy is usually variable and depends on the site and extent of infection as well as the clinical condition of the patient (63–66, 68–70). Antimicrobial therapy is usually given for 3 months or even much longer duration particularly in disseminated infections. At times, medical therapy may be prolonged and repeated cycles of antimicrobial therapy may be required in recurrent or partially treated infections (63–66, 68–70).

Surgical treatment in the form of debridement may be needed in severe localized infections such as tunnel infections as well as serious skin and soft tissue infections (64). However, additional therapies such as growth factors and non-steroidal anti-inflammatory drugs may be given to augment immunity of patients having NTM infections. Nevertheless, such additional therapies may not be effective (69).

Cessation or tapering of immunosuppressive therapies given to recipients of allogeneic HSCT, particularly those having GVHD, may facilitate treatment of NTM infections. However, such maneuver may also be ineffective (69).

## Interactions between HSCT Therapies and Antimicrobials

Treatment of NTM disease in transplant recipients may be complicated by the possibility of drug interactions between antimicrobial agents and immunosuppressive therapies and the possibility of allograft rejection or worsening of GVHD as a result of reduction in immunosuppression during NTM treatment (1). Drug interactions between immunosuppressive agents such as cyclosporine-A and tacrolimus and various antimicrobials given to control NTM infections are real concerns as they have significant impact not only on control of the underlying infectious process but also on the outcome of HSCT (69, 74–76).

Cyclosporine-A is an immunosuppressive agent that is frequently used in allogeneic HSCT to prevent GVHD. It is metabolized by cytochrome P-450 3A4 (CYP 3A4) in hepatic system. Antimicrobials that induce or inhibit CYP enzymes and that are concurrently administered with cyclosporine-A are likely to alter blood levels of cyclosporine-A (74). Thus, drugs that increase blood levels of cyclosporine-A are likely to potentiate its side effects particularly nephrotoxicity, while antimicrobials that decrease blood levels of cyclosporine-A are likely to cause graft rejection (75). Examples of antimicrobials that alter blood levels of cyclosporine-A include (1) macrolides such as ciprofloxacin, (2) cephalosporins such as cefepime, (3) azoles such as fluconazole, and (4) TMP–SMZ (74, 75).

Tacrolimus is being increasingly used as an immunosuppressant agent in recipients of SOT and HSCT. It is metabolized by CYP 3A4 system, thus, antimicrobials that inhibit or induce these enzymes can alter blood levels of tacrolimus (76). Antimicrobials that increase blood levels of tacrolimus will further aggravate its nephrotoxicity and neurotoxicity, while antimicrobials, which decrease blood levels of tacrolimus, are likely to increase the risk of organ or graft rejection (76). The following antimicrobials that are used in the treatment of NTM infections have been reported to interact with tacrolimus (1) macrolides such as clarithromycin and azithromycin, (2) aminoglycosides such as tobramycin, amikacin, and streptomycin, (3) quinolones such as ofloxacin and ciprofloxacin, (4) anti-mycobacterial agents such as rifampin, rifabutin, ethambutol, and pyrazinamide, and (5) other antimicrobials such as aztreonam, meropenem, sulfonamides, and tetracyclines (76). Therefore, it is essential to (1) modify doses of antimicrobials as well as immunosuppressive therapies as needed and (2) have rigorous monitoring of not only adverse effects but also drug levels of medications used in HSCT recipients (75).

## Course and Prognosis of NTM Infections in HSCT

The course of NTM disease in recipients of HSCT may be prolonged, persistent, and may take the form of remissions and exacerbations. Significant morbidity and mortality has been reported particularly in patients having other comorbid medical conditions (26, 64, 69–71). Good outcome is associated with: appropriate antimicrobial therapy, CVC-related infections and localized disease while poor prognosis is associated with: inappropriate antimicrobial therapy, delayed initiation of antibiotic treatment, disseminated disease, bloodstream infections, and severe immunosuppression in the host (63–67, 69–72).

## Conclusion

Infections caused by NTM are increasingly recognized in immunocompromised individuals including patients with cancer and recipients of SOT as well as HCST. Both slowly growing and rapidly growing NTM species cause variable infectious complications in recipients of almost all forms of HSCT. These infections carry significant morbidity and mortality. Prompt diagnosis and early initiation of appropriate antimicrobials therapy improve the outcome of NTM infections in HSCT recipients.

## Conflict of Interest Statement

The authors declare that the research was conducted in the absence of any commercial or financial relationships that could be construed as a potential conflict of interest.
